# Selected mutli-drug-resistance-organisms in selected Polish long-term-care facilities*

**DOI:** 10.1186/1753-6561-5-S6-P160

**Published:** 2011-06-29

**Authors:** J Wojkowska-Mach, D Romaniszyn, M Pobiega, B Gryglewska, T Grodzicki

**Affiliations:** 1 Jagiellonian University Medical School, Kraków, Poland

## Introduction / objectives

Nosocomial infections and antimicrobial resistance (AR) are a very well-known public health problems. Unfortunately, up to now there is no surveillance of AR in Polish long term care facilities (LTCF). The aim of this presentation was to analyze prevalence of selected Multi-Drug-Resistance-Organisms (MDRO) among residents of LTCFs in Krakow, Poland.

## Methods

The study was carried out from 10-2009 to 11-2010 in 3 public LTCF on a group of 193 residents. Screening test for MRSA and ESBL(+) Gram-negative rods were done at the beginning of the study. Also urine and wound cultures were performed, together with microbiological examinations of any biological materials taken when symptoms of infection were observed.

## Results

Altogether 28.9% of the residents had wheelchair disability or were bedridden, 10.6% - disoriented in time and/or space, 49,5% had urinary incontinence and 35,3% fecal incontinence. Prevalence of MDRO was found to be 12.4%. The most common pathogens were MRSA, which consisted 43,2% of all *S. aureus* isolates (n=94) and ESBL(+) rods, which reached 13,9% out of 165 *Enterobacteriaceae* isolates. Factors independently (all p<0.05) associated with MDRO were asymptomatic bacteriuria, urinary and/or fecal incontinence, wheelchair disability or bedridden, low value of the Barthel and Katz Indexes and others.

## Conclusion

This study showed that the prevalence of MRSA and ESBL(+) rods was lower then in other similar studies published recently. Unfortunately, microbiology tests were done not in all cases of infections which occurred during the study like as sputum samples from suspected pneumonia (14,3%). Thus, results obtained by us, importance of infection control for elderly patients. * This study was supported by the grant from the Ministry of Science and Higher Education (N N404 047236).

## Disclosure of interest

None declared.

